# Bis(tri-2-pyridyl­amine)­nickel(II) bis­(perchlorate)

**DOI:** 10.1107/S1600536810051627

**Published:** 2010-12-15

**Authors:** Shi Wang, Wenrui He, Wei Huang

**Affiliations:** a School of Materials Science & Engineering, Nanjing University of Posts and Telecommunications, Nanjing 210046, People’s Republic of China

## Abstract

In the title compound, [Ni(C_15_H_12_N_4_)_2_](ClO_4_)_2_, the Ni^II^ atom lies on an inversion center and is octa­hedrally coordinated by the N atoms of two tridentate tri-2-pyridyl­amine ligands. The two perchlorate anions are disordered over two sites with a refined occupancy ratio of 0.528 (19):0.472 (19).

## Related literature

For background to luminescent coordination compounds, see: Liu *et al.* (1997 ▶). For related complexes, including the synthesis of 2,2′,2′′-tpa (tpa is tri-2-pyridylamine), see: Yang *et al.* (1999 ▶). For information on the use of 2,2′,2′′-tpa as a bidentate ligand, see: Wang *et al.* (2009 ▶). 
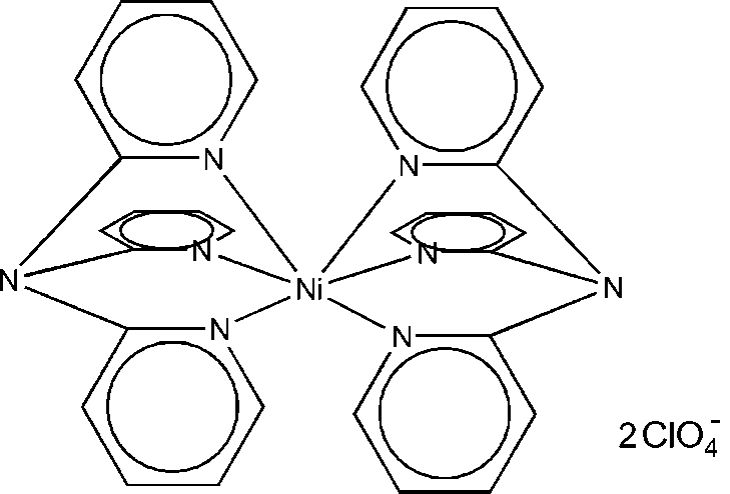


## Experimental

### 

#### Crystal data

[Ni(C_15_H_12_N_4_)_2_](ClO_4_)_2_
*M* *_r_* = 754.18
Monoclinic, 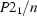

*a* = 8.360 (4) Å
*b* = 17.570 (8) Å
*c* = 11.165 (5) Å
β = 99.542 (5)°
*V* = 1617.3 (13) Å^3^
*Z* = 2
Mo *K*α radiation
μ = 0.83 mm^−1^
*T* = 296 K
0.22 × 0.15 × 0.10 mm

#### Data collection

Bruker SMART CCD area-detector diffractometer
Absorption correction: multi-scan (*SADABS*; Sheldrick, 1996 ▶) *T*_min_ = 0.861, *T*_max_ = 0.920
14055 measured reflections
3895 independent reflections
2611 reflections with *I* > 2σ(*I*)
*R*_int_ = 0.040

#### Refinement

*R*[*F* ^2^ > 2σ(*F* ^2^)] = 0.039
*wR*(*F* ^2^) = 0.096
*S* = 1.03
3895 reflections
269 parameters
H-atom parameters constrained
Δρ_max_ = 0.25 e Å^−3^
Δρ_min_ = −0.26 e Å^−3^

### 

Data collection: *SMART* (Bruker, 2007 ▶); cell refinement: *SAINT* (Bruker, 2007 ▶); data reduction: *SAINT*; program(s) used to solve structure: *SHELXTL* (Sheldrick, 2008 ▶); program(s) used to refine structure: *SHELXTL*; molecular graphics: *SHELXTL*; software used to prepare material for publication: *SHELXTL*.

## Supplementary Material

Crystal structure: contains datablocks I, global. DOI: 10.1107/S1600536810051627/nk2079sup1.cif

Structure factors: contains datablocks I. DOI: 10.1107/S1600536810051627/nk2079Isup2.hkl

Additional supplementary materials:  crystallographic information; 3D view; checkCIF report

## Figures and Tables

**Fig. 1.**
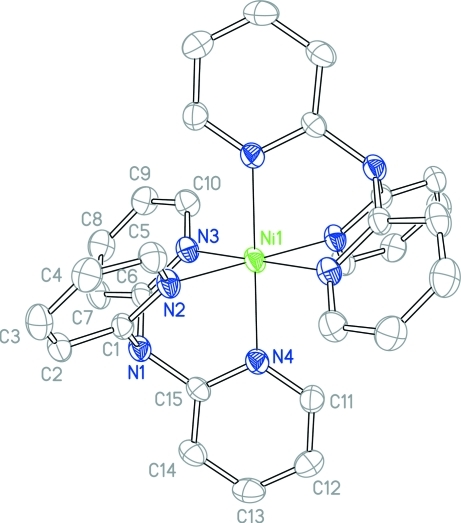
The structure of (I), shown with 30% probability displacement ellipsoids. The disordered perchlorate anions and H atoms have been omitted for clarity. Unlabeled atoms are related to labeled atoms by inversion symmetry.

## supplementary crystallographic information

### Comment

Luminescent organic and coordination compounds have been an active research area for decades because of their various potential applications in materials sciences. It has been demonstrated that 2,2'-dipyridylamine can produce a bright blue luminescence when deprotonated and bound to either an aluminium ion or a boron center (Liu *et al.*, 1997). However, many of the previously reported aluminium or boron compounds based on 2,2'-dipyridylamine are not stable enough for electroluminescent devices. The neutral tripodal ligand 2,2',2''-tripyridylamine and its derivatives with Zn(II), Cd(II) and Hg(II) have been investigated for their optical properties (Yang *et al.*, 1999). However, its complexes with d^8^ metal(II) remain unkown. By using the 2,2',2''-tripyridylamine ligand, we report here the synthesis and crystal structure of the title compound, bis(2,2',2''-tripyridylamine)nickel(II) bis(perchlorate), (I).

The structure of (I) consists of monomeric [Ni(2,2',2''-tpa)_2_]^2+^ cations and associated ClO_4_^-^ anions. The Ni atom lies on an inversion center. As shown in Fig. 1, the Ni center is six-coordinate with an octahedral geometry. In principle, 2,2',2''-tpa can function not only as a tridentate chelating ligand but also as a bidentate chelating ligand where only two pyridyl groups bind to the same central atom (Wang *et al.*, 2009). In the title compound, each 2,2',2''-tpa ligand functions as a tridentate ligand, chelating to the nickel center. The two perchlorate anions are disordered over two sites with a refined occupancy ratio of 0.528 (19) : 0.472 (19).

### Experimental

The ligand, 2,2',2''-tpa was synthesized according to the procedure described in the literature (Yang *et al.* (1999)).

A solution of 2,2',2''-tpa (62.11 mg, 0.2 mmol) in acetonitrile (5 ml) was added dropwise to a solution of Ni(ClO_4_)_2_.6H_2_O (36.58 mg, 0.1 mmol) in acetonitrile (2 ml). The mixture was stirred at room temperature for 5 min and then filtered. Light purple crystals of (I) suitable for X-ray analysis were obtained by slow diffusion of filtrate.

### Refinement

The two perchlorate anions are disordered over two sites with a refined occupancy ratio of 0.528 (19) : 0.472 (19). Aromatic H atoms were placed in calculated positions with C—H = 0.93 Å, and refined in riding mode with *U*_iso_(H) = 1.2Ueq(C).

### Crystal data

**Table:**

[Ni(C_15_H_12_N_4_)_2_](ClO_4_)_2_	*F*(000) = 772
*M**_r_* = 754.18	*D*_x_ = 1.549 Mg m^−^^3^
Monoclinic, *P*2_1_/*n*	Mo *K*α radiation, λ = 0.71073 Å
Hall symbol: -P 2yn	Cell parameters from 7662 reflections
*a* = 8.360 (4) Å	θ = 2.2–27.9°
*b* = 17.570 (8) Å	µ = 0.83 mm^−^^1^
*c* = 11.165 (5) Å	*T* = 296 K
β = 99.542 (5)°	Block, purple
*V* = 1617.3 (13) Å^3^	0.22 × 0.15 × 0.10 mm
*Z* = 2	

### Data collection

**Table:**

Bruker SMART CCD area-detector diffractometer	3895 independent reflections
Radiation source: fine-focus sealed tube	2611 reflections with *I* > 2σ(*I*)
graphite	*R*_int_ = 0.040
Detector resolution: 8.366 pixels mm^-1^	θ_max_ = 28.4°, θ_min_ = 2.3°
phi and ω scans	*h* = −11→11
Absorption correction: multi-scan (*SADABS*; Sheldrick, 1996)	*k* = −23→23
*T*_min_ = 0.861, *T*_max_ = 0.920	*l* = −14→14
14055 measured reflections	

### Refinement

**Table:**

Refinement on *F*^2^	Primary atom site location: structure-invariant direct methods
Least-squares matrix: full	Secondary atom site location: difference Fourier map
*R*[*F*^2^ > 2σ(*F*^2^)] = 0.039	Hydrogen site location: inferred from neighbouring sites
*wR*(*F*^2^) = 0.096	H-atom parameters constrained
*S* = 1.03	*w* = 1/[σ^2^(*F*_o_^2^) + (0.0415*P*)^2^ + 0.1545*P*] where *P* = (*F*_o_^2^ + 2*F*_c_^2^)/3
3895 reflections	(Δ/σ)_max_ = 0.001
269 parameters	Δρ_max_ = 0.25 e Å^−^^3^
0 restraints	Δρ_min_ = −0.26 e Å^−^^3^

### Special details

**Table:**

Geometry. All esds (except the esd in the dihedral angle between two l.s. planes) are estimated using the full covariance matrix. The cell esds are taken into account individually in the estimation of esds in distances, angles and torsion angles; correlations between esds in cell parameters are only used when they are defined by crystal symmetry. An approximate (isotropic) treatment of cell esds is used for estimating esds involving l.s. planes.
Refinement. Refinement of F^2^ against ALL reflections. The weighted R-factor wR and goodness of fit S are based on F^2^, conventional R-factors R are based on F, with F set to zero for negative F^2^. The threshold expression of F^2^ > 2sigma(F^2^) is used only for calculating R-factors(gt) etc. and is not relevant to the choice of reflections for refinement. R-factors based on F^2^ are statistically about twice as large as those based on F, and R- factors based on ALL data will be even larger.

### Fractional atomic coordinates and isotropic or equivalent isotropic displacement parameters (Å^2^)

**Table:**

	*x*	*y*	*z*	*U*_iso_*/*U*_eq_	Occ. (<1)
Ni1	0.0000	0.0000	0.0000	0.03843 (14)	
N1	0.1260 (2)	−0.05805 (10)	−0.21770 (15)	0.0415 (4)	
N2	0.1529 (2)	−0.09211 (10)	−0.00942 (15)	0.0425 (4)	
N3	−0.1341 (2)	−0.04164 (10)	−0.16286 (16)	0.0425 (5)	
N4	0.1399 (2)	0.05822 (10)	−0.10841 (16)	0.0428 (4)	
C1	0.1923 (3)	−0.10685 (12)	−0.11845 (19)	0.0398 (5)	
C2	0.2910 (3)	−0.16666 (13)	−0.1379 (2)	0.0491 (6)	
H2	0.3180	−0.1751	−0.2143	0.059*	
C3	0.3487 (3)	−0.21360 (14)	−0.0420 (2)	0.0588 (7)	
H3	0.4148	−0.2548	−0.0528	0.071*	
C4	0.3080 (3)	−0.19915 (13)	0.0696 (2)	0.0546 (6)	
H4	0.3456	−0.2305	0.1354	0.066*	
C5	0.2114 (3)	−0.13810 (13)	0.0829 (2)	0.0496 (6)	
H5	0.1852	−0.1281	0.1591	0.060*	
C6	−0.0476 (3)	−0.06409 (12)	−0.24704 (19)	0.0414 (5)	
C7	−0.1176 (3)	−0.09311 (14)	−0.3578 (2)	0.0547 (6)	
H7	−0.0542	−0.1076	−0.4150	0.066*	
C8	−0.2834 (4)	−0.10013 (15)	−0.3818 (2)	0.0647 (7)	
H8	−0.3339	−0.1197	−0.4559	0.078*	
C9	−0.3738 (3)	−0.07818 (14)	−0.2965 (2)	0.0577 (7)	
H9	−0.4861	−0.0832	−0.3110	0.069*	
C10	−0.2954 (3)	−0.04864 (14)	−0.1890 (2)	0.0533 (6)	
H10	−0.3572	−0.0327	−0.1317	0.064*	
C11	0.1900 (3)	0.13045 (13)	−0.0929 (2)	0.0492 (6)	
H11	0.1612	0.1583	−0.0290	0.059*	
C12	0.2822 (3)	0.16462 (14)	−0.1682 (2)	0.0575 (7)	
H12	0.3156	0.2149	−0.1552	0.069*	
C13	0.3246 (3)	0.12403 (16)	−0.2624 (2)	0.0627 (7)	
H13	0.3886	0.1461	−0.3136	0.075*	
C14	0.2716 (3)	0.05020 (14)	−0.2809 (2)	0.0527 (6)	
H14	0.2970	0.0219	−0.3456	0.063*	
C15	0.1808 (3)	0.01931 (12)	−0.20188 (19)	0.0402 (5)	
Cl1	0.2377 (6)	0.3455 (4)	0.0694 (3)	0.0463 (11)	0.472 (19)
O1	0.1538 (19)	0.3916 (9)	0.1111 (12)	0.128 (5)	0.472 (19)
O2	0.224 (3)	0.2727 (6)	0.1240 (8)	0.153 (7)	0.472 (19)
O3	0.3932 (15)	0.3751 (8)	0.1296 (13)	0.129 (4)	0.472 (19)
O4	0.214 (2)	0.3370 (9)	−0.0534 (9)	0.140 (6)	0.472 (19)
Cl1'	0.2323 (10)	0.3362 (6)	0.0668 (6)	0.095 (2)	0.528 (19)
O1'	0.1418 (16)	0.4114 (7)	0.0767 (15)	0.133 (5)	0.528 (19)
O2'	0.1138 (18)	0.2869 (8)	0.0907 (17)	0.169 (5)	0.528 (19)
O3'	0.3760 (15)	0.3273 (15)	0.1348 (13)	0.205 (9)	0.528 (19)
O4'	0.2693 (18)	0.3279 (7)	−0.0504 (11)	0.109 (4)	0.528 (19)

### Atomic displacement parameters (Å^2^)

**Table:**

	*U*^11^	*U*^22^	*U*^33^	*U*^12^	*U*^13^	*U*^23^
Ni1	0.0488 (3)	0.0391 (2)	0.0291 (2)	0.00336 (19)	0.01177 (17)	−0.00443 (17)
N1	0.0529 (12)	0.0401 (10)	0.0331 (10)	0.0029 (9)	0.0114 (9)	−0.0027 (8)
N2	0.0543 (12)	0.0416 (10)	0.0319 (10)	0.0059 (9)	0.0082 (8)	−0.0038 (8)
N3	0.0476 (12)	0.0456 (11)	0.0343 (10)	0.0033 (9)	0.0069 (9)	−0.0043 (8)
N4	0.0525 (12)	0.0421 (10)	0.0355 (10)	−0.0008 (9)	0.0125 (9)	−0.0026 (8)
C1	0.0457 (13)	0.0387 (12)	0.0355 (11)	0.0002 (10)	0.0079 (10)	−0.0066 (9)
C2	0.0524 (15)	0.0475 (13)	0.0496 (14)	0.0036 (11)	0.0149 (12)	−0.0110 (11)
C3	0.0608 (17)	0.0466 (14)	0.0683 (17)	0.0154 (12)	0.0091 (14)	−0.0037 (13)
C4	0.0587 (16)	0.0474 (14)	0.0553 (15)	0.0077 (12)	0.0022 (13)	0.0065 (12)
C5	0.0603 (16)	0.0517 (14)	0.0369 (13)	0.0048 (12)	0.0080 (11)	0.0005 (11)
C6	0.0542 (15)	0.0386 (12)	0.0321 (11)	0.0019 (10)	0.0092 (10)	−0.0034 (9)
C7	0.0695 (18)	0.0591 (15)	0.0352 (13)	0.0035 (13)	0.0073 (12)	−0.0110 (11)
C8	0.075 (2)	0.0696 (18)	0.0440 (15)	−0.0041 (15)	−0.0063 (14)	−0.0125 (13)
C9	0.0534 (16)	0.0610 (16)	0.0549 (16)	−0.0016 (13)	−0.0021 (13)	−0.0030 (13)
C10	0.0530 (16)	0.0580 (15)	0.0489 (14)	0.0027 (12)	0.0082 (12)	−0.0058 (12)
C11	0.0578 (16)	0.0439 (13)	0.0462 (14)	−0.0016 (11)	0.0095 (12)	−0.0046 (11)
C12	0.0610 (17)	0.0502 (14)	0.0608 (17)	−0.0084 (13)	0.0091 (13)	0.0075 (13)
C13	0.0621 (17)	0.0682 (18)	0.0627 (17)	−0.0046 (14)	0.0242 (14)	0.0156 (14)
C14	0.0595 (16)	0.0600 (16)	0.0426 (14)	0.0065 (13)	0.0199 (12)	0.0062 (11)
C15	0.0434 (13)	0.0465 (13)	0.0317 (11)	0.0049 (10)	0.0095 (10)	0.0010 (9)
Cl1	0.046 (2)	0.072 (2)	0.0231 (16)	0.0105 (14)	0.0106 (12)	−0.0077 (12)
O1	0.158 (8)	0.140 (10)	0.099 (5)	0.080 (7)	0.058 (6)	0.001 (6)
O2	0.31 (2)	0.084 (6)	0.070 (5)	−0.006 (9)	0.037 (8)	0.018 (4)
O3	0.095 (6)	0.186 (10)	0.100 (6)	−0.052 (7)	0.000 (4)	−0.031 (6)
O4	0.195 (12)	0.199 (9)	0.023 (4)	0.091 (8)	0.013 (5)	−0.005 (4)
Cl1'	0.109 (4)	0.101 (3)	0.075 (3)	0.037 (3)	0.014 (3)	−0.020 (2)
O1'	0.107 (5)	0.070 (5)	0.211 (13)	0.027 (4)	−0.004 (6)	−0.036 (6)
O2'	0.169 (9)	0.122 (7)	0.251 (13)	0.007 (7)	0.139 (9)	0.013 (7)
O3'	0.086 (8)	0.36 (3)	0.148 (8)	0.120 (11)	−0.033 (7)	−0.089 (13)
O4'	0.144 (8)	0.120 (6)	0.080 (6)	−0.018 (6)	0.068 (6)	−0.032 (4)

### Geometric parameters (Å, °)

**Table:**

Ni1—N2	2.0755 (19)	C7—C8	1.373 (4)
Ni1—N2^i^	2.0755 (19)	C7—H7	0.9300
Ni1—N4	2.0851 (18)	C8—C9	1.367 (4)
Ni1—N4^i^	2.0851 (18)	C8—H8	0.9300
Ni1—N3^i^	2.1029 (19)	C9—C10	1.370 (3)
Ni1—N3	2.1029 (19)	C9—H9	0.9300
N1—C15	1.436 (3)	C10—H10	0.9300
N1—C1	1.437 (3)	C11—C12	1.370 (3)
N1—C6	1.438 (3)	C11—H11	0.9300
N2—C5	1.337 (3)	C12—C13	1.366 (4)
N2—C1	1.338 (3)	C12—H12	0.9300
N3—C6	1.337 (3)	C13—C14	1.375 (3)
N3—C10	1.337 (3)	C13—H13	0.9300
N4—C15	1.339 (3)	C14—C15	1.368 (3)
N4—C11	1.339 (3)	C14—H14	0.9300
C1—C2	1.375 (3)	Cl1—O1	1.212 (13)
C2—C3	1.374 (3)	Cl1—O4	1.361 (11)
C2—H2	0.9300	Cl1—O2	1.430 (13)
C3—C4	1.369 (3)	Cl1—O3	1.457 (12)
C3—H3	0.9300	Cl1'—O3'	1.320 (11)
C4—C5	1.366 (3)	Cl1'—O2'	1.375 (14)
C4—H4	0.9300	Cl1'—O4'	1.401 (11)
C5—H5	0.9300	Cl1'—O1'	1.537 (15)
C6—C7	1.375 (3)		
			
N2—Ni1—N2^i^	180.0	N3—C6—C7	122.9 (2)
N2—Ni1—N4	86.78 (7)	N3—C6—N1	117.46 (18)
N2^i^—Ni1—N4	93.22 (7)	C7—C6—N1	119.7 (2)
N2—Ni1—N4^i^	93.22 (7)	C8—C7—C6	118.3 (2)
N2^i^—Ni1—N4^i^	86.78 (7)	C8—C7—H7	120.9
N4—Ni1—N4^i^	180.0	C6—C7—H7	120.9
N2—Ni1—N3^i^	94.05 (7)	C9—C8—C7	119.7 (2)
N2^i^—Ni1—N3^i^	85.95 (7)	C9—C8—H8	120.1
N4—Ni1—N3^i^	93.52 (8)	C7—C8—H8	120.1
N4^i^—Ni1—N3^i^	86.48 (8)	C8—C9—C10	118.5 (3)
N2—Ni1—N3	85.95 (7)	C8—C9—H9	120.7
N2^i^—Ni1—N3	94.05 (7)	C10—C9—H9	120.7
N4—Ni1—N3	86.48 (8)	N3—C10—C9	123.0 (2)
N4^i^—Ni1—N3	93.52 (8)	N3—C10—H10	118.5
N3^i^—Ni1—N3	180.0	C9—C10—H10	118.5
C15—N1—C1	113.30 (17)	N4—C11—C12	122.2 (2)
C15—N1—C6	112.77 (17)	N4—C11—H11	118.9
C1—N1—C6	112.15 (17)	C12—C11—H11	118.9
C5—N2—C1	118.06 (19)	C13—C12—C11	119.2 (2)
C5—N2—Ni1	125.59 (15)	C13—C12—H12	120.4
C1—N2—Ni1	116.34 (14)	C11—C12—H12	120.4
C6—N3—C10	117.54 (19)	C12—C13—C14	119.3 (2)
C6—N3—Ni1	115.96 (15)	C12—C13—H13	120.3
C10—N3—Ni1	126.49 (15)	C14—C13—H13	120.3
C15—N4—C11	118.01 (19)	C15—C14—C13	118.5 (2)
C15—N4—Ni1	116.26 (15)	C15—C14—H14	120.8
C11—N4—Ni1	125.72 (15)	C13—C14—H14	120.8
N2—C1—C2	122.4 (2)	N4—C15—C14	122.8 (2)
N2—C1—N1	117.66 (18)	N4—C15—N1	117.50 (18)
C2—C1—N1	119.90 (19)	C14—C15—N1	119.74 (19)
C3—C2—C1	118.5 (2)	O1—Cl1—O4	117.6 (10)
C3—C2—H2	120.7	O1—Cl1—O2	109.8 (9)
C1—C2—H2	120.7	O4—Cl1—O2	108.8 (9)
C4—C3—C2	119.3 (2)	O1—Cl1—O3	96.5 (10)
C4—C3—H3	120.3	O4—Cl1—O3	118.3 (10)
C2—C3—H3	120.3	O2—Cl1—O3	104.7 (9)
C5—C4—C3	119.0 (2)	O3'—Cl1'—O2'	115.5 (11)
C5—C4—H4	120.5	O3'—Cl1'—O4'	101.8 (10)
C3—C4—H4	120.5	O2'—Cl1'—O4'	113.2 (9)
N2—C5—C4	122.6 (2)	O3'—Cl1'—O1'	118.1 (12)
N2—C5—H5	118.7	O2'—Cl1'—O1'	98.6 (8)
C4—C5—H5	118.7	O4'—Cl1'—O1'	110.1 (9)
			
N4—Ni1—N2—C5	138.49 (19)	C2—C3—C4—C5	−0.3 (4)
N4^i^—Ni1—N2—C5	−41.51 (19)	C1—N2—C5—C4	−0.4 (3)
N3^i^—Ni1—N2—C5	45.18 (19)	Ni1—N2—C5—C4	178.09 (17)
N3—Ni1—N2—C5	−134.82 (19)	C3—C4—C5—N2	0.9 (4)
N4—Ni1—N2—C1	−42.98 (16)	C10—N3—C6—C7	0.4 (3)
N4^i^—Ni1—N2—C1	137.02 (16)	Ni1—N3—C6—C7	179.54 (18)
N3^i^—Ni1—N2—C1	−136.29 (16)	C10—N3—C6—N1	−178.68 (19)
N3—Ni1—N2—C1	43.71 (16)	Ni1—N3—C6—N1	0.4 (2)
N2—Ni1—N3—C6	−44.10 (16)	C15—N1—C6—N3	−65.0 (2)
N2^i^—Ni1—N3—C6	135.90 (16)	C1—N1—C6—N3	64.4 (2)
N4—Ni1—N3—C6	42.92 (16)	C15—N1—C6—C7	115.9 (2)
N4^i^—Ni1—N3—C6	−137.08 (16)	C1—N1—C6—C7	−114.8 (2)
N2—Ni1—N3—C10	134.9 (2)	N3—C6—C7—C8	−0.9 (4)
N2^i^—Ni1—N3—C10	−45.1 (2)	N1—C6—C7—C8	178.2 (2)
N4—Ni1—N3—C10	−138.1 (2)	C6—C7—C8—C9	0.2 (4)
N4^i^—Ni1—N3—C10	41.9 (2)	C7—C8—C9—C10	0.8 (4)
N2—Ni1—N4—C15	43.27 (16)	C6—N3—C10—C9	0.7 (3)
N2^i^—Ni1—N4—C15	−136.73 (16)	Ni1—N3—C10—C9	−178.30 (18)
N3^i^—Ni1—N4—C15	137.13 (16)	C8—C9—C10—N3	−1.3 (4)
N3—Ni1—N4—C15	−42.87 (16)	C15—N4—C11—C12	−0.8 (3)
N2—Ni1—N4—C11	−137.4 (2)	Ni1—N4—C11—C12	179.82 (18)
N2^i^—Ni1—N4—C11	42.6 (2)	N4—C11—C12—C13	0.1 (4)
N3^i^—Ni1—N4—C11	−43.5 (2)	C11—C12—C13—C14	1.0 (4)
N3—Ni1—N4—C11	136.5 (2)	C12—C13—C14—C15	−1.5 (4)
C5—N2—C1—C2	−0.6 (3)	C11—N4—C15—C14	0.3 (3)
Ni1—N2—C1—C2	−179.22 (17)	Ni1—N4—C15—C14	179.77 (18)
C5—N2—C1—N1	178.7 (2)	C11—N4—C15—N1	−179.87 (19)
Ni1—N2—C1—N1	0.1 (3)	Ni1—N4—C15—N1	−0.4 (3)
C15—N1—C1—N2	64.0 (3)	C13—C14—C15—N4	0.8 (4)
C6—N1—C1—N2	−65.1 (2)	C13—C14—C15—N1	−179.0 (2)
C15—N1—C1—C2	−116.7 (2)	C1—N1—C15—N4	−63.5 (3)
C6—N1—C1—C2	114.2 (2)	C6—N1—C15—N4	65.3 (2)
N2—C1—C2—C3	1.1 (3)	C1—N1—C15—C14	116.3 (2)
N1—C1—C2—C3	−178.2 (2)	C6—N1—C15—C14	−115.0 (2)
C1—C2—C3—C4	−0.6 (4)		

Symmetry codes: (i) −*x*, −*y*, −*z*.
